# Tomato Puree Enrichment in a High‐Fat Meal Reduces Postprandial Plasma and Adipose Tissue Inflammation Biomarkers in Healthy Male Adults: A Crossover Randomized Controlled Trial

**DOI:** 10.1002/mnfr.70115

**Published:** 2025-05-12

**Authors:** Lea Sani, Julien Astier, Djaffar Ould‐Ali, Patrick Borel, Jean‐François Landrier

**Affiliations:** ^1^ Aix Marseille Univ., C2VN, INRAE, INSERM Marseille France; ^2^ CHU La Conception, AP‐HM Marseille France

**Keywords:** adipose tissue, gene expression, inflammatory biomarkers, lycopene, postprandial inflammation, tomato puree

## Abstract

The postprandial period is marked by increased plasma inflammatory biomarkers and heightened adipose tissue inflammation. Consumption of tomato‐based products has been linked to reduced inflammation, potentially lowering the risk of cardiometabolic diseases. This study investigated the addition of tomato puree to a high‐fat meal (HFM) on postprandial inflammation in healthy men. Thirty‐nine healthy men participated in a randomized crossover trial (NCT02100774). Two meals were given: a HFM and the same HFM with 100 g tomato puree (TPM). Blood samples and adipose tissue biopsies were taken at various timepoints to measure inflammatory markers. TPM reduced plasma tumor necrosis factor alpha (TNF‐α), interleukin (IL)‐6, and C‐C motif chemokine ligand (CCL)‐2, and gene expression of *TNFA*, *IL6*, *IL1B*, and *CCL5* in adipose tissue. In contrast, HFM increased IL‐1β, CCL2, and adipose gene expressions of *IL6*, *IL1B*, and *CCL2*. Variations and net incremental area under the curve (iAUCs_net_) between groups showed significantly lower inflammatory markers in TPM, except for plasma CCL2 and *CCL5* expression. Incorporating tomato puree in HFM reduces both systemic and adipose tissue inflammation during the postprandial period. These findings suggest that tomato‐based products may contribute to the reduction of postprandial inflammation, potentially explaining their cardiometabolic benefits.

## Graphical abstract

Tomato puree enrichment in the context of a high‐fat meal in healthy adult males leads to a significant reduction of systemic postprandial and adipose tissue postprandial inflammation.

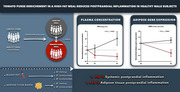

**Abbreviations**
BMI: body mass index
CCL: C‐C motif chemokine ligand
CRP: C Reactive protein
HFM: high‐fat meal
HIV: human immunodeficiency virus
HPLC: high performance liquid chromatography
iAUC_net_
: net incremental area under the curve
IGF‐1R: insulin‐like growth factor‐1 receptor
IL: interleukin
IQR: interquartile range
IκB: IkappaB Kinase
JNK: Jun N‐terminal Kinase
LPS: lipopolysaccharide
MUFA: monounsatured fatty acids
NCDs: non‐communicable diseases
NF‐κB: nuclear factor‐kappa B
PUFA: polyunsatured fatty acids
RNA: ribonucleic acid
ROS: reactive oxygen species
rRNA: ribosomal ribonucleic acid
SFA: saturated fatty acids
SP1: stimulatory Protein‐1
T2DM: type 2 Diabetes Mellitus
TNF‐α: tumor necrosis factor alpha
TPM: tomato puree meal
WHO: World Health Organization

## 1 Introduction

Inflammation is a primary physiological response of tissues to injury, leading to the activation of the immune system. The ingestion of a meal also leads to a complex endocrine, metabolic, and immune response, resulting in a temporary inflammatory state as the body works to restore balance [1, 2]. Western diets, high in calories, fat, and carbohydrates, are characterized by postprandial metabolic imbalance, leading to long‐term low‐grade inflammation, hyperlipidemia, and hyperglycemia, which play a role in the development of non‐communicable diseases (NCDs), such as obesity, type 2 diabetes (T2DM) and cardiovascular diseases [3, 4].

Adipose tissue is an important endocrine organ [5], containing adipocytes and other cells (preadipocytes and immune cells) that secrete a variety of hormones, acute phase proteins, cytokines, and chemokines. Postprandial low‐grade inflammation activates signaling pathways such as jun N‐terminal kinase (JNK) and nuclear factor‐kappa B (NF‐κB), notably in adipose tissue, leading to the secretion of pro‐inflammatory markers such as tumor necrosis factor‐alpha (TNF‐α), interleukin (IL) 6, IL‐1β, C‐C motif chemokine ligand (CCL) 2, and CCL‐5 [6]. During the postprandial state, cytokine levels increase dose‐dependently around 2 to 3 h and then return to normal levels by 8 h postingestion [6].

The Mediterranean diet, characterized by a high intake of fruits and vegetables, is associated with a reduction of inflammation due to the anti‐inflammatory and antioxidant properties of its components [7]. Several epidemiological studies have shown that this diet is inversely correlated with the onset of NCDs [8, 9, 10, 11]. Tomato is one of the most popular fruits and the second most consumed vegetable in the world [12], and is rich in phytomicronutrients known for their health benefits. Minerals are abundant in tomatoes, with potassium being the most prominent (353 mg per 100 g), followed by phosphorus, calcium, and magnesium. Tomatoes are also rich in vitamins, particularly vitamin C (18.9 mg per 100 g), vitamin E, and various B vitamins. Phenolic compounds such as quercetin, kaempferol, and naringenin are present, contributing to the tomato's antioxidant properties. The carotenoid content of tomatoes is notable, with lycopene being the primary carotenoid responsible for the fruit's red color [13]. Tomato processing (cooking, drying, etc.) induces isomerization of *all‐trans* lycopene (predominant natural form), changing its conformation to *cis*‐lycopene, which increases bioavailability [14]. Lycopene is known for its antioxidant properties and constitutes one of the predominant carotenoids in human plasma [15]. It exhibits an anti‐inflammatory action in adipocytes and liver by inhibiting the production of pro‐inflammatory cytokines through the NF‐κB pathway, specifically by modulating IKKα/β phosphorylation [5, 16–18]. Several epidemiological studies have revealed a negative correlation between tomato consumption, plasma lycopene levels, and the risk of developing cancers and NCDs [13, 15, 19]. Daily consumption of tomato, tomato‐based products, as well as lycopene supplementation over periods ranging from 1 week to 6 months, has been shown to reduce biomarkers of oxidative stress and inflammation in healthy individuals [20, 21, 22, 23, 24, 25]. This may contribute to the prevention of the development of cardiometabolic diseases [26]. Only two studies have investigated the effect of tomato or tomato product consumption on postprandial inflammation and reported similar effects [27, 28], but none have yet investigated the combined effect on inflammatory gene expressions in adipose tissue and plasma concentrations. Evidence is lacking in this regard, and it is necessary to focus first on the short‐term effects of consuming lycopene‐rich products to expect to understand the long‐term effects [26]. Accordingly, the present study was designed to assess the impact of high lycopene tomato puree enrichment in a high‐fat meal (HFM) on postprandial inflammation in healthy male volunteers.

## 2 Methods

### 2.1 Ethical Statement

Ethical approval for the involvement of human subjects in this study was granted by the CCP Ouest I Ethics Committee (France), Reference number (no. 2008‐A01354‐51) and registered in ClinicalTrials (NCT02100774). The study was conducted according to the guidelines laid down in the Declaration of Helsinki of 1975 as revised in 1983 and to the guidelines for Good Clinical Practice of ICH.

### 2.2 Subjects

Forty healthy male volunteers aged between 18 and 60 were included. Eligibility of participants required to be male, nonsmokers in good general health with a body mass index (BMI) between 19 and 24 kg/m^2^. Because of the relatively large volume of blood collected during the study, a blood hemoglobin concentration >8.0 mmol/L was also an inclusion criterion. The subjects recruited had no dyslipidemia or hyperglycemia and were negative for the human immunodeficiency virus (HIV) and hepatitis C and B. Therefore, criteria for non‐inclusion included fasting cholesterol concentration <6.47 mmol/L, fasting triglyceride concentration <2.26 mmol/L, fasting blood glucose ≤6.05 mmol/L, vegetarian diet, regular use of vitamin supplements, alcohol consumption <140 g/week (<2 glasses of alcohol/day), history of chronic disease, presence of any disease, and/or use of drugs known to affect lipid metabolism or fat‐soluble micronutrients during the month before the study (e.g., ezetimibe). One subject left the study for personal reasons before he participated in the postprandial experiment, which left 39 subjects.

### 2.3 Study Design

The present trial was a controlled, crossover, conducted at the Clinal Investigation Center of the Conception Hospital (Hopital de la Conception; Marseille, France). Subjects were randomly allocated into two groups using a random number table. To ensure impartiality, the allocation process was conducted by an investigator who was not clinically engaged in the study. Both researchers and participants were kept unaware of the randomization and allocation until the completion of data analyses. Upon arrival at the Conception Hospital, each group presented in a fasted state and received two distinct meal types, described below, by their randomized allocation: a HFM, characterized by a fat content exceeding established recommendations (>40% of total daily energy intake), or an enriched HFM, comprising the same HFM but supplemented with 100 g of tomato puree meal (TPM). The two types of meals were consumed on two separate visits, separated by a washout period of at least 3 weeks, which allowed the total excretion of the compounds from the first meal, in order to ensure that the observations from the second meal were not influenced by the previous one.

Subjects were asked to refrain from consuming vitamin supplements and lycopene‐rich foods (an exclusion list was provided by a dietitian) for 48 h before the postprandial experiment. The day before the postprandial experiment, they were also asked to dine between 7:00 p.m. and 8:00 p.m., without any alcohol intake, and to abstain from consuming any food or beverage other than water after dinner and until the clinic visit. After the overnight fast, they arrived at the clinic and consumed the test meal.

The HFM consisted of semolina cooked (70 g) in 200 mL of hot water, white bread (40 g), cooked egg whites (60 g), peanut oil (50 g), and mineral water (330 mL). In this meal, the energy contribution of lipids exceeds 40% of the total energy intake, which justifies the HFM designation, in line with the recommendations of the World Health Organization (WHO), which recommends not exceeding 30% of total daily energy intake. Peanut oil was selected for its relatively neutral nutritional profile compared with other oils. Its fatty acid profile favors monounsatured fatty acids (MUFA) and polyunsatured fatty acids (PUFA) (omega 6 type), it is low in saturated fatty acids (SFA) and contains reduced levels of vitamin E compared with other oils. However, its inclusion in the meal results in a SFA intake of 11.27% of total energy intake, exceeding the 10% limit recommended by the WHO. It is also easy to incorporate into a meal, due to its neutral taste and its widespread use for cooking and seasoning in many cultures around the world. The TPM was identical but supplemented with 100 g of tomato puree purchased from a local supermarket. The composition of the HFM was determined using the table of nutritional composition of foods (CIQUAL) [29]. The global composition of the tomato puree was determined by the nutritional label product and the carotenoid composition by high‐performance liquid chromatography (HPLC) as previously reported [30]. Table 1 details the nutritional composition of the food components of the HFM and TPM.

**TABLE 1:** Macronutrient and micronutrient composition of meals administered in the study.

Nutritional composition^a^	Energy (kcal)	Proteins (g)	Carbohydrates (g)	Fat (g)	Fibers (g)	Lycopene (mg)
Semolina^b^ (70 g)	85.4	2.4	16.8	0.5	1.3	0
Egg whites^b^ (60 g)	28.4	6.2	0.7	0.1	0	0
White bread (40 g)	102.4	3.1	20.8	0.4	1.1	0
Peanut oil (50 g)	450	0	0	50.0	0	0
Tomato puree (100 g)	47.1	1.4	7.5	0.4	2.4	10.2
HFM^c^, ^d^	666.2	11.7	38.3	51.0	2.4	0.0
TPM^e^, ^f^	713.3	13.1	45.8	51.4	4.8	10.2

*Note*: The global composition of the tomato puree was determined by the nutritional label product, and the carotenoid composition by high performance liquid chromatography (HPLC); 100 g of tomato puree provided 9.7 mg all‐trans lycopene and 0.5 mg cis isomers of lycopene.

^a^ HFM, high fat meal; TPM, tomato puree meal.

^b^ Values for cooked ingredients.

^c^ The HFM designation is justified by the energy contribution of lipids exceeding 40% of total energy intake.

^d^ Total nutritional composition for the HFM, which contains all foods except tomato puree. Nutritional analysis by conversion of data from the French food composition table ANSES—Ciqual.

^e^ The TPM was identical to the HFM meal, with the addition of 100 g of tomato puree.

^f^ Total nutritional composition for the TPM which contains all foods including tomato puree.

Meal intake was controlled, and subjects were asked to consume the meal at a regular rate, consuming half of the meal in about 15 min and the rest of the meal in 30 min to minimize the variability caused by the different rates of ingestion and thus gastric emptying. Over the next 8 h, participants abstained from food, although they were allowed to drink the remaining water from the meal. Blood samples were collected at baseline 0 h, 2 h, and 8 h, and a biopsy was performed at 0 and 8 h after each meal.

### 2.4 Biochemical Measures

Blood samples were collected via evacuated purple‐top glass tubes containing potassium‐EDTA. Tubes were immediately placed on ice and protected from light. Plasma was obtained by centrifugation (1620 × *g* for 10 min at 4°C) and was stored at –80°C. Biochemical analysis was performed at the Cardiovascular and Nutrition Research Center (C2VN).

### 2.5 Measurement of Basal Plasma Glucose and Lipids

Serum triglycerides, total cholesterol, and glucose were determined by enzymatic procedures with commercial kits (Boehringer). Hemoglobin concentrations were measured with a calibrated laboratory machine (ADVIA 2120 hematology system, Siemens Healthcare) immediately after blood sample collection.

### 2.6 Measurement of Plasma Pro‐Inflammatory Cytokines and Chemokines

The concentration of pro‐inflammatory cytokines and chemokines TNF‐α, IL‐6, IL‐1β, and CCL2 were determined by enzyme‐linked immunosorbent procedures, using a commercial Human ELISA kit (eBioscience). The kits were used according to the manufacturer's instructions.

### 2.7 Subcutaneous Adipose Tissue Collection

Subcutaneous adipose tissue (∼100–200 mg) was biopsied from the periumbilical region by suction with a sterile 20‐mL syringe in which a vacuum was previously drawn, after local anesthesia (2% non‐adrenalized xylocaine). Tissue samples were stored in free RNA tubes at –80°C for gene expression analyses.

### 2.8 Measurement of the Expression of Inflammation Genes

Total RNA was extracted from the biopsies and stored at –80°C using the reagent TRIzol (Invitrogen), according to the manufacturer's manual. Real‐time quantitative RT‐PCR analyses were performed using the Mx3005P Real‐Time PCR System (Stratagene; Amsterdam, The Netherlands) as previously described [31], using SYBR green kits (Eurogentec; Angers, France), according to the manufacturer instructions. Expression was quantified in duplicate for *TNFA, IL6, IL1B, CCL2*, and *CCL5* genes, and 18S rRNA was used as the endogenous control in the comparative cycle threshold (CT) method [32]. Results were expressed as relative expression ratio (mRNA/18S rRNA).

### 2.9 Calculations

Postprandial inflammation was quantified by calculating the net incremental area under the curve (iAUC_net_) of plasma inflammatory marker concentration and inflammatory gene expression relative to collection time for each participant after each supplementation using the trapezoidal rule. The iAUC_net_ was calculated by subtracting each plasma concentration or gene expression value at time 2 h or 8 h by the respective subject's baseline value.

### 2.10 Statistical Analysis and Data Management

Normality and homogeneity of variance of data were assessed by the Shapiro‐Wilk test and the Brown‐Forsythe test, respectively. Outliers were identified using the Tukey's method [33]. Values falling outside 1.5 times the interquartile range (IQR) below the first quartile or above the third quartile were eliminated. Each subject served as his own control; therefore, the following tests were performed on paired data.

Two types of analysis were performed; intra‐group analysis and inter‐group analysis. The intra‐group analysis consisted of a comparison between each collection time (0 h, 2 h, and 8 h) in each group of meals (HFM and TPM). For plasma data, this analysis was performed using repeated measures ANOVA, and post hoc Tukey tests were used for two‐by‐two comparisons of times in each meal (0 h vs. 2 h; 2 h vs. 8, and 0 h vs. 8 h). For gene expression data, this analysis was performed using a paired *t*‐test (0 h vs. 8 h). The Greenhouse‐Geisser correction was applied when the sphericity of the data could be assumed.

The inter‐group analysis included a comparison of iAUCs_net_ using a paired *t*‐test and a generalized linear model (GLM) was used to assess the differences in variations (Δ = parameter value at 8 h—parameter value at 0 h) in plasma concentrations and gene expression in adipose tissue of inflammatory markers (Δ_8h–0h_) between the HFM and TPM groups. An ANOVA analysis was subsequently used to examine the group effect in this model. The GLM model was adjusted for parameters with a possible influence (BMI, age, order of administration of different meals). A Wald test demonstrated the null effect of each covariate on the dependent variable, and a likelihood ratio test showed that the addition of these covariates did not influence the results of the ANOVA analysis.

Quantitative variables were represented as mean ± SEM for tables and as mean ± SEM for graphs.

The linear relationship of variations between plasma concentrations and gene expression levels in adipose tissue of inflammatory markers, for each meal (TPM and HFM) and each marker, was assessed by linear regression models. The coefficient of determination (*R* ^2^) and *p* value (*p*) were calculated for each analysis, and the regression coefficient (*β*) was additionally calculated for the linear regressions.

Statistical analyses were performed using Prism (version 9.4.0) and R (version 4.4.1.). A statistically significant value of *p* < 0.05 was used.

## 3 Results

### 3.1 Subject Baseline Characteristics and Meal Nutritional Profiles

Of the 39 volunteers included, all individuals completed the study. The basal characteristics of the subjects are shown in Table 2.

**TABLE 2:** Table of basal characteristics of the subjects.

Parameters	Mean ± SEM (Reference ranges)
Age (y)	32 ± 2
Weight (kg)	72.4 ± 1.3
BMI (kg/m^2^)	22.7 ± 0.3 (18.5–24.9)
Hemoglobin (mmol/L)^a^	9.37 ± 0.1 (8.7–11.2)
Glucose (mmol/L)^a^	4.73 ± 0.07 (3.9–6.1)
Triglycerides (mmol/L)^a^	0.92 ± 0.07 (0.5–2.0)
Total cholesterol (mmol/L)^a^	4.31 ± 0.15 (<5.2)

^a^ Fasting plasma concentrations.

The population had a mean age ± SEM (minimum; maximum) of 32 ± 2 years (19; 59). They weighed 73.0 ± 8.2 kg and had a BMI of 22.9 ± 2.1 kg/m^2^. The detailed inclusion criteria demonstrate that the population was not affected by disturbances in glucose and lipid homeostasis. Subjects reported normoenergetic consumption (i.e., approximately 2500 kcal/d) with <2% alcohol as total daily energy intake.

In agreement with Table 1, the HFM was slightly lower in energy (−7.1%; 666.2 vs. 713.3 kcal), protein (−11.7%; 11.9 vs. 13.1 g), carbohydrate (−19.6%; 38.3 vs. 45.8 g), and fiber (−99.2%; 2.4 vs. 4.8 g) than the TPM. Previous HPLC measurements revealed that the tomato puree contained 9.7 mg *all‐trans* lycopene and approximately 0.5 mg *cis* lycopene for TPM, compared with an absence of lycopene in the HFM [30].

### 3.2 Impact of Tomato Puree Enrichment on Postprandial Plasma Inflammatory Markers

The results of the intra‐group analysis for plasma inflammatory markers are reported in Table 3.

**TABLE 3:** Intra‐group comparison analysis^a^ of plasma concentrations of inflammatory markers (fasting vs. postprandial).

HFM						TPM				
	Repeated measures ANOVA^b^, ^d^			Tukey's HSD post‐hoc test^c^, ^d^		Repeated measures ANOVA^b^, ^d^			Tukey's HSD post‐hoc test^c^, ^d^	
	Time	Plasma concentration (pg/mL) (mean ± SEM)	*p* value	Time	*p* value	Time	Plasma concentration (pg/mL) (mean ± SEM)	*p* value	Time	*p* value
**TNF‐**α	**0 h**	79.2 ± 21.4	*0.1378*	**0 h vs. 2 h**	*0.8560*	**0 h**	44.3 ± 8.0	***0.0160***	**0 h vs. 2 h**	*0.0691*
	**2 h**	83.9 ± 21.2		**2 h vs. 8 h**	*0.3522*	**2 h**	30.4 ± 3.4		**2 h vs. 8 h**	*0.8536*
	**8 h**	110.0 ± 32.7		**0 h vs. 8 h**	*0.2177*	**8 h**	27.2 ± 4.0		**0 h vs. 8 h**	***0.0184***
**IL‐6**	**0 h**	5.96 ± 1.28	***0.0994***	**0 h vs. 2 h**	*0.7867*	**0 h**	4.98 ± 0.97	***0.0448***	**0 h vs. 2 h**	***0.0356***
	**2 h**	6.39 ± 1.14		**2 h vs. 8 h**	*0.3092*	**2 h**	2.93 ± 0.24		**2 h vs. 8 h**	*0.2984*
	**8 h**	7.37 ± 1.21		**0 h vs. 8 h**	*0.0904*	**8 h**	4.14 ± 0.40		**0 h vs. 8 h**	*0.5560*
**IL‐1**β	**0 h**	16.0 ± 1.74	***0.0034***	**0 h vs. 2 h**	***0.0023***	**0 h**	22.9 ± 2.54	*0.2368*	**0 h vs. 2 h**	*0.9982*
	**2 h**	19.7 ± 1.72		**2 h vs. 8 h**	*0.1221*	**2 h**	23.1 ± 1.23		**2 h vs. 8 h**	*0.2208*
	**8 h**	17.6 ± 1.92		**0 h vs. 8 h**	*0.2884*	**8 h**	19.4 ± 1.67		**0 h vs. 8 h**	*0.3320*
**CCL‐2**	**0 h**	237.1 ± 40.4	***0.0146***	**0 h vs. 2 h**	***0.0104***	**0 h**	236.7 ± 30.4	***0.0104***	**0 h vs. 2 h**	***0.0214***
	**2 h**	284.8 ± 41.5		**2 h vs. 8 h**	*0.2559*	**2 h**	386.8 ± 56.7		**2 h vs. 8 h**	***0.0246***
	**8 h**	259.5 ± 44.2		**0 h vs. 8 h**	*0.3420*	**8 h**	239.7 ± 18.8		**0 h vs. 8 h**	*0.9984*

^a^ Stratified comparison (HFM and TPM) of the plasma concentrations (pg/mL) of inflammatory markers at baseline 0 h, 2 h, and 8 h after ingestion of HFM or TPM.

^b^ Intra‐group comparison was performed using a repeated measures ANOVA. The Greenhouse‐Geisser correction was applied when the sphericity of the data could be assumed.

^c^ Posthoc analyses were performed with all ANOVA tests using a Tuckey Honest Significant Difference test to compare each collection time two‐by‐two. Tukey‐adjusted *p* values for post hoc tests of pairwise differences are reported.

^d^ *p* < 0.05 indicates a significant difference, highlighted in bold.

Plasma TNF‐α concentrations were significantly different between collection times for the TPM in contrast to the HFM (*p *= 0.0160 vs. *p *= 0.1378). Indeed, post‐hoc analysis showed a 38.6% decrease in TNF‐α concentration between 0 and 8 h (*p *= 0.0184) for TPM. Plasma IL‐6 concentrations were not significantly different for the HFM, contrary to the TPM (*p *= 0.0994 vs. *p *= 0.0448), with a decrease of 41.2% between baseline and 2 h (*p *= 0.0356) for TPM. Plasma IL‐1β concentrations were not significantly different for the TPM, whereas they were for the HFM (*p *= 0.2368 vs. *p *= 0.0034), with an increase of 23.1% between baseline and 2 h (*p *= 0.0023). Plasma CCL2 concentrations were different for the TPM and HFM (*p *= 0.0104 vs. *p *= 0.0146), with an increase of 63.4% between baseline and 2 h (*p *= 0.0214) and a decrease of 38.0% between 2 and 8 h (*p *= 0.0246) for TPM and an increase of 20.1% between baseline and 2 h (*p *= 0.0104) for HFM.

**FIGURE 1:**
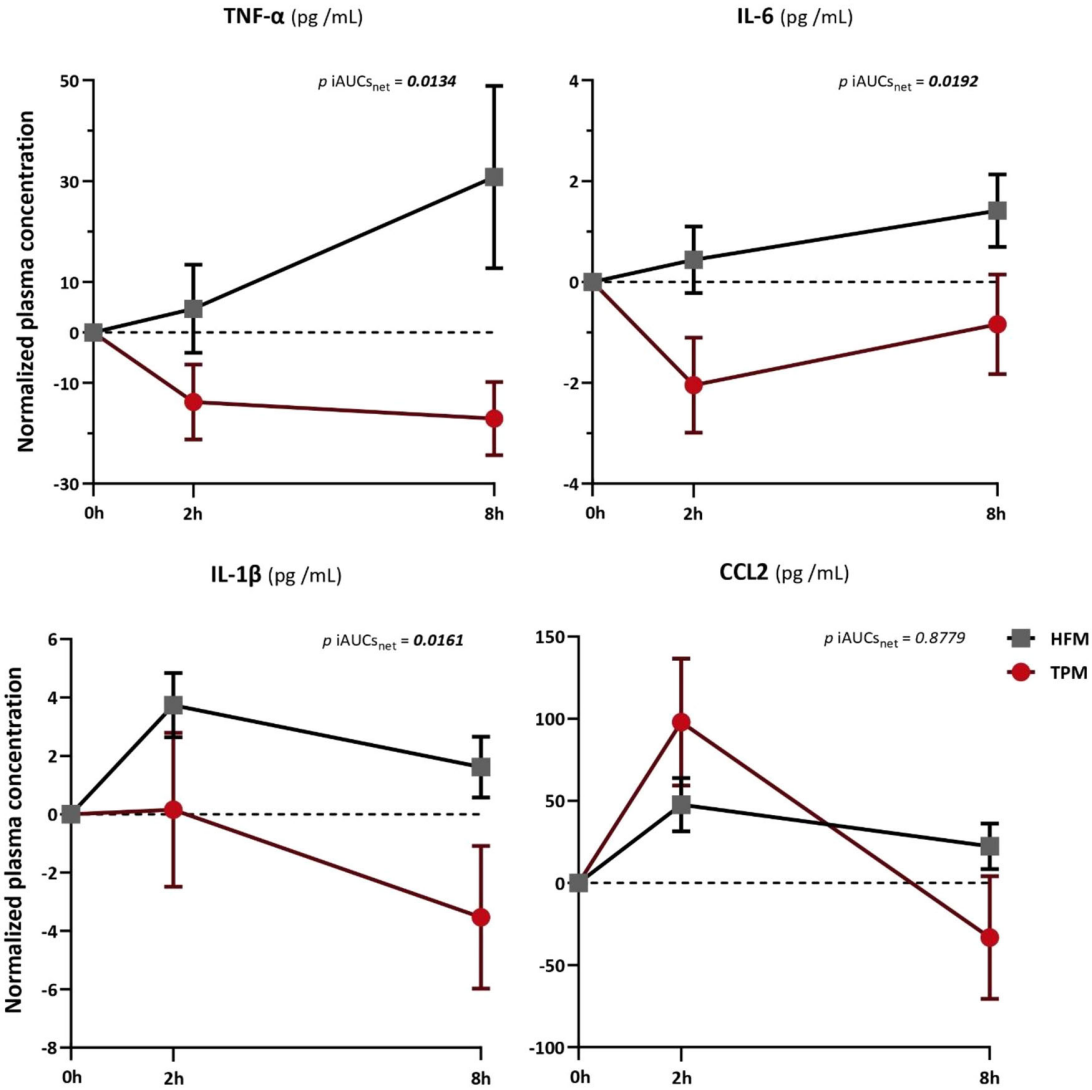
Inter‐group comparison analysis of iAUCs_net_ of postprandial plasma concentrations of inflammatory markers (TPM vs. HFM). Mean ± SEM (biological replicates) of normalized plasma concentrations (postprandial values—fasting value). *p* values correspond to inter‐group comparisons of mean iAUCs_net_ between TPM (red) (*n *= 39) and HFM (grey) (*n *= 39) groups, using a paired *t*‐test, and *p* < 0.05 indicates a significant difference, highlighted in bold. CCL2, C‐C motif chemokine ligand 2; iAUC_net_, net incremental area under the curve; HFM, high fat meal; IL‐1β, interleukin 1 beta; IL‐6, interleukin 6; TNF‐α, tumor necrosis factor alpha; TPM, tomato puree meal.

Figure 1 shows the evolution of normalized plasma concentrations of inflammatory markers as a function of time, and the inter‐group comparison of the postprandial iAUCs_net_ between TPM and HFM.

For plasma concentrations of the markers TNF‐α, IL‐6, and IL‐1β, the iAUCs_net_ for the HFM were positive (above dotted‐0 axis) in contrast to the TPM, which were negative (below dotted‐0 axis). The iAUCs_net_ for the CCL2 marker was positive for both meals. The iAUCs_net_ of TNF‐α, IL‐6 and IL‐1β plasma markers in the TPM showed significant and respective reductions (mean ± SEM, *p* value of iAUCs_net_ comparison) of 195.2% (−106.6 ± 45.7 vs. ± 112.0 ± 64.3, *p *= 0.0134), 277.8% (−10.7 ± 6.7 vs. 6.0 ± 4.0, *p *= 0.0192) and 174.6% (−17.4 ± 15.0 vs. 23.4 ± 5.8, *p *= 0.0161), compared with the HFM. The iAUCs_net_ of the CCL2 plasma marker was not different between the TPM and HFM (381.0 ± 218.7 vs. 258.3 ± 88.7, *p *= 0.8779).

**FIGURE 2:**
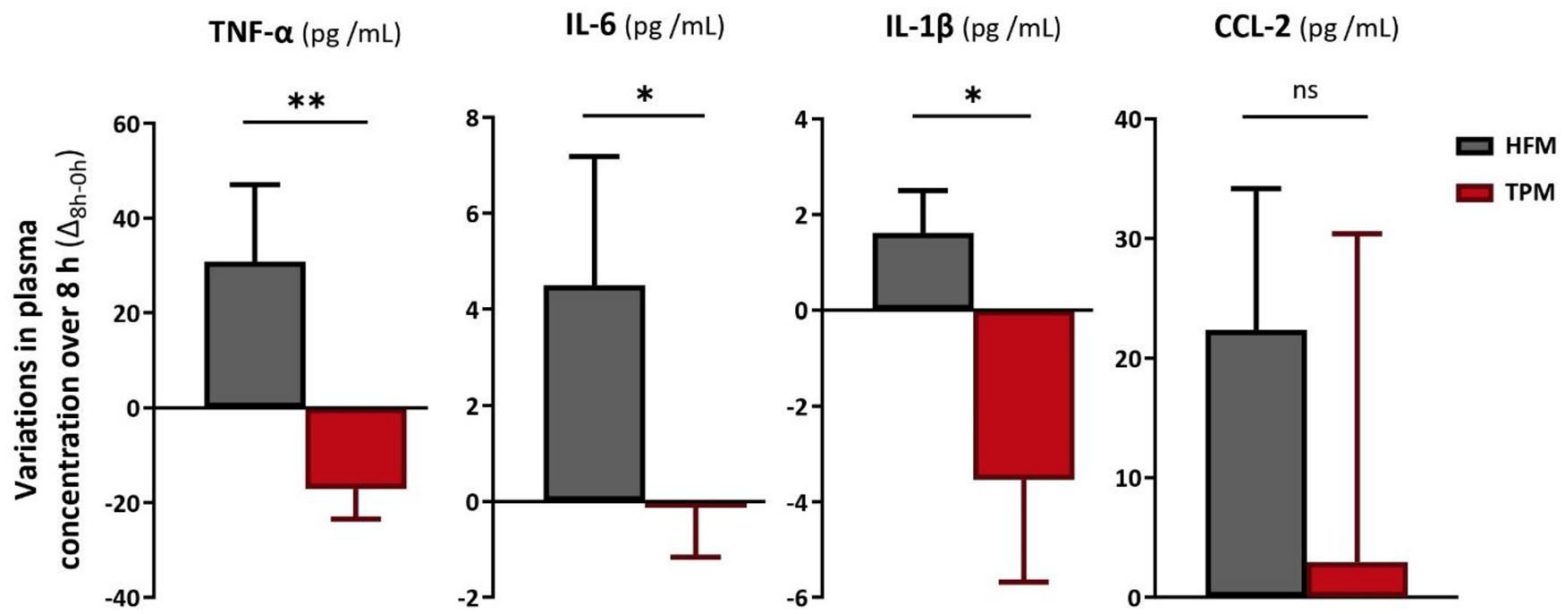
Inter‐group comparative analysis of postprandial variations in plasma concentrations of inflammatory markers (TPM vs. HFM). Mean ± SEM (biological replicates) of changes (Δ) in plasma concentrations between baseline (0 h) and postprandial (8 h). *p* values correspond to the ANOVA analysis of the GLM model that compared variations between TPM (red) (*n *= 39) and HFM (grey) (*n *= 39) groups; *p* < 0.05 indicates a significant difference. The difference between groups is indicated by *: *p* < 0.05; **: *p* < 0.01. The variation was calculated between each collection time (Δ_8h–0h_), according to the following calculation: (postprandial value—fasting value). CCL2, C‐C motif chemokine ligand 2; HFM, high fat meal; IL‐1β, interleukin 1 beta; IL‐6, interleukin 6; TNF‐α, tumor necrosis factor alpha; TPM, tomato puree meal.

The GLM inter‐group comparison of the postprandial variations in plasma concentrations of inflammatory markers between the TPM and HFM is presented in Figure 2.

Firstly, variations in plasma concentrations at different sampling times were predominantly positive (to the right of axis 0) for HFM, while those for TPM were predominantly negative (to the left of axis 0). Consequently, plasma concentrations of inflammatory markers tended to increase postprandially for HFM and decrease for TPM. The plasma concentrations of the TNF‐α marker showed a significant reduction of 155.5% for variations Δ_8h–0h_ (−17.1 ± 6.4 vs. 30.8 ± 16.3 pg/mL, *p *= 0.0080). The variation in plasma concentration of the inflammatory marker IL‐6 was significantly decreased in the TPM, compared with the HFM, by 103.0% for variations Δ_8h–0h_ (−0.1 ± 1.0 vs. 4.5 ± 2.7 pg/mL, *p *= 0.0317). Regarding the inflammatory marker IL‐1β, plasma concentration variations were significantly reduced in the TPM, compared with the HFM, by 319.3% for variations Δ_8h‐0 h_ (−3.5 ± 2.1 vs. 1.6 ± 0.9 pg/mL, *p *= 0.0349). For inflammatory marker CCL‐2, plasma concentration variations were not significantly reduced in TPM, compared with HFM, by 86.9% for variations Δ_8h–0 h_ (2.9 ± 27.5 vs. 22.4 ± 11.8 pg/mL, *p *= 0.5317).

### 3.3 Impact of Tomato Puree Enrichment on Postprandial Expression of Adipose Tissue Inflammatory Gene

The results of the intra‐group analysis for adipose tissue inflammatory gene expression are reported in Table 4.

**TABLE 4:** Intra‐group comparison analysis^a^, ^b^ of gene expression of inflammatory markers in adipose tissue (fasting vs. postprandial).

	HFM			TPM		
	Paired *t*‐test^c^			Paired *t*‐test^c^		
	Time	Gene expression (mRNA/18S rRNA) (mean ± SEM)	*p* value	Time	Gene expression (mRNA/18S rRNA) (mean ± SEM)	*p* value
***TNFA***	**0 h**	88.6 ± 7.43	*0.2466*	**0 h**	102.8 ± 14.1	***0.0071***
	**8 h**	101.4 ± 9.52		**8 h**	62.3 ± 7.03	
***IL6***	**0 h**	68.9 ± 7.92	***0.0240***	**0 h**	105.1 ± 14.6	***0.0229***
	**8 h**	221.2 ± 62.8		**8 h**	70.5 ± 6.86	
***IL1B***	**0 h**	87.6 ± 8.01	***0.0006***	**0 h**	99.8 ± 15.2	***0.0019***
	**8 h**	150.9 ± 17.6		**8 h**	54.1 ± 4.53	
***CCL2***	**0 h**	93.3 ± 9.53	***0.0375***	**0 h**	116.1 ± 18.3	*0.2493*
	**8 h**	169.2 ± 35.9		**8 h**	96.3 ± 7.81	
***CCL5***	**0 h**	79.5 ± 12.7	*0.8176*	**0 h**	108.8 ± 21.1	***0.0419***
	**8 h**	75.8 ± 17.4		**8 h**	69.5 ± 5.54	

^a^ Stratified comparison (HFM and TPM) of gene expression (mRNA/18S rRNA) of inflammatory markers in adipose tissue at baseline 0 h and 8 h after ingestion of HFM or TPM.

^b^ Intra‐group comparison was performed using a paired *t*‐test.

^c^ *p* < 0.05 indicates a significant difference, highlighted in bold.

Between 0 and 8 h, *TNFA* gene expression was significantly decreased by 39.4% in the TPM (*p *= 0.0071) and non‐significantly increased by 14.5% in the HFM (*p *= 0.2466). *IL6* gene expression between 0 and 8 h was significantly decreased by 32.9% for the TPM (*p *= 0.0229) and significantly increased by 221.1% for the HFM (*p *= 0.0240). *IL1B* gene expression between 0 and 8 h significantly decreased by 45.8% for TPM (*p *= 0.0019) and significantly increased by 72.3% for HFM (*p *= 0.0006). *CCL2* gene expression between 0 and 8 h was non‐significantly decreased by 17.1% for the TPM (*p *= 0.2493) and significantly increased by 81.4% for HFM (*p *= 0.0375). *CCL5* gene expression between 0 and 8 h decreased significantly by 36.1% for the TPM (*p *= 0.0419) and non‐significantly by 4.7% for the HFM (*p *= 0.8176).

**FIGURE 3:**
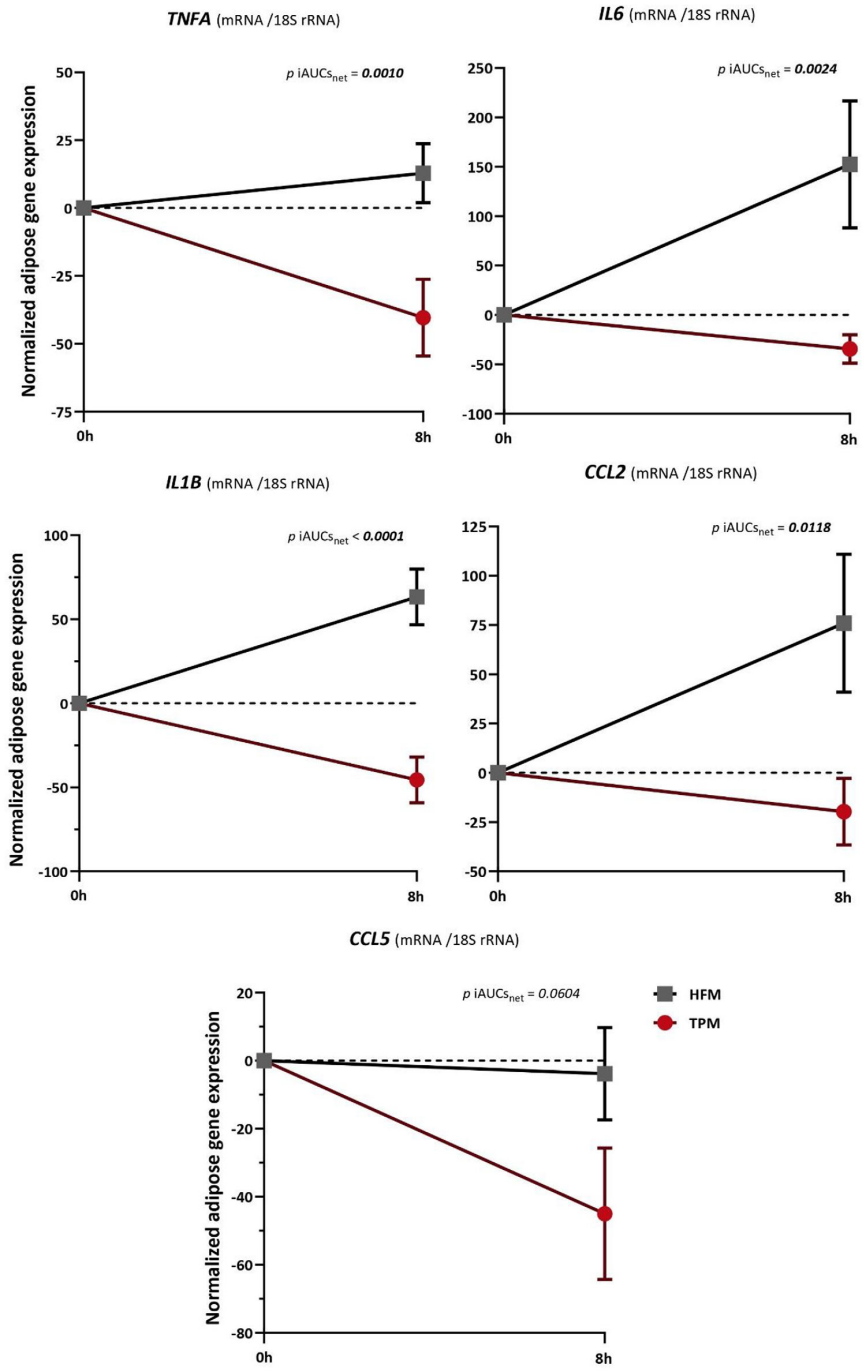
Inter‐group comparison analysis of iAUCs_net_ of postprandial gene expression of inflammatory markers in adipose tissue (TPM vs. HFM). Mean ± SEM (Biological replicates) of gene expression levels (postprandial value—fasting value). *p* values correspond to inter‐group comparisons of mean iAUCs_net_ between TPM (red) (*n *= 39) and HFM (grey) (*n *= 39) groups, using a paired *t*‐test, and *p* < 0.05 indicates a significant difference, highlighted in bold. *CCL2*, C‐C motif chemokine ligand 2, *CCL5*, C‐C motif chemokine ligand 5 gene, HFM, high fat meal, iAUC_net_, net incremental area under the curve, *IL1B*, interleukin 1 beta, *IL6*, interleukin 6, *TNFA*, tumor necrosis factor alpha, TPM, tomato puree meal.

Figure 3 shows the evolution of normalized adipose tissue inflammatory gene expression as a function of time, and the inter‐group comparison of the postprandial iAUCs_net_ between TPM and HFM.

For expression of all adipose tissue inflammatory genes except *CCL5*, the iAUCs_net_ of the HFM were positive (above dotted‐0 axis) in contrast to the TPM, which was negative (below dotted‐0 axis). The inter‐group comparison of iAUCs_net_ of adipose gene expression of *TNFA, IL6, IL1B*, and *CCL2* in the TPM showed significant respective reductions compared with the HFM (mean ± SEM, *p* value of iAUCs_net_ comparison) of 414.6% (−151.2 ± 56.2 vs. ±74.6 ± 37.6, *p *= 0.0010, 122.7% (−138.4 ± 55.0 vs. 609.5 ± 230.0, *p *= 0.0024), 172.1% (−182.6 ± 54.7 vs. 253.4 ± 59.3, *p *< 0.0001) and 126.1% (−79.1 ± 65.7 vs. 303.7 ± 121.3, *p *= 0.0118). The iAUCs_net_ of adipose gene expression of *CCL5* in the TPM did not show a significant difference compared with the HFM (−179.8 ± 73.1 vs. −11.2 ± 46.6, *p *= 0.0604).

**FIGURE 4:**
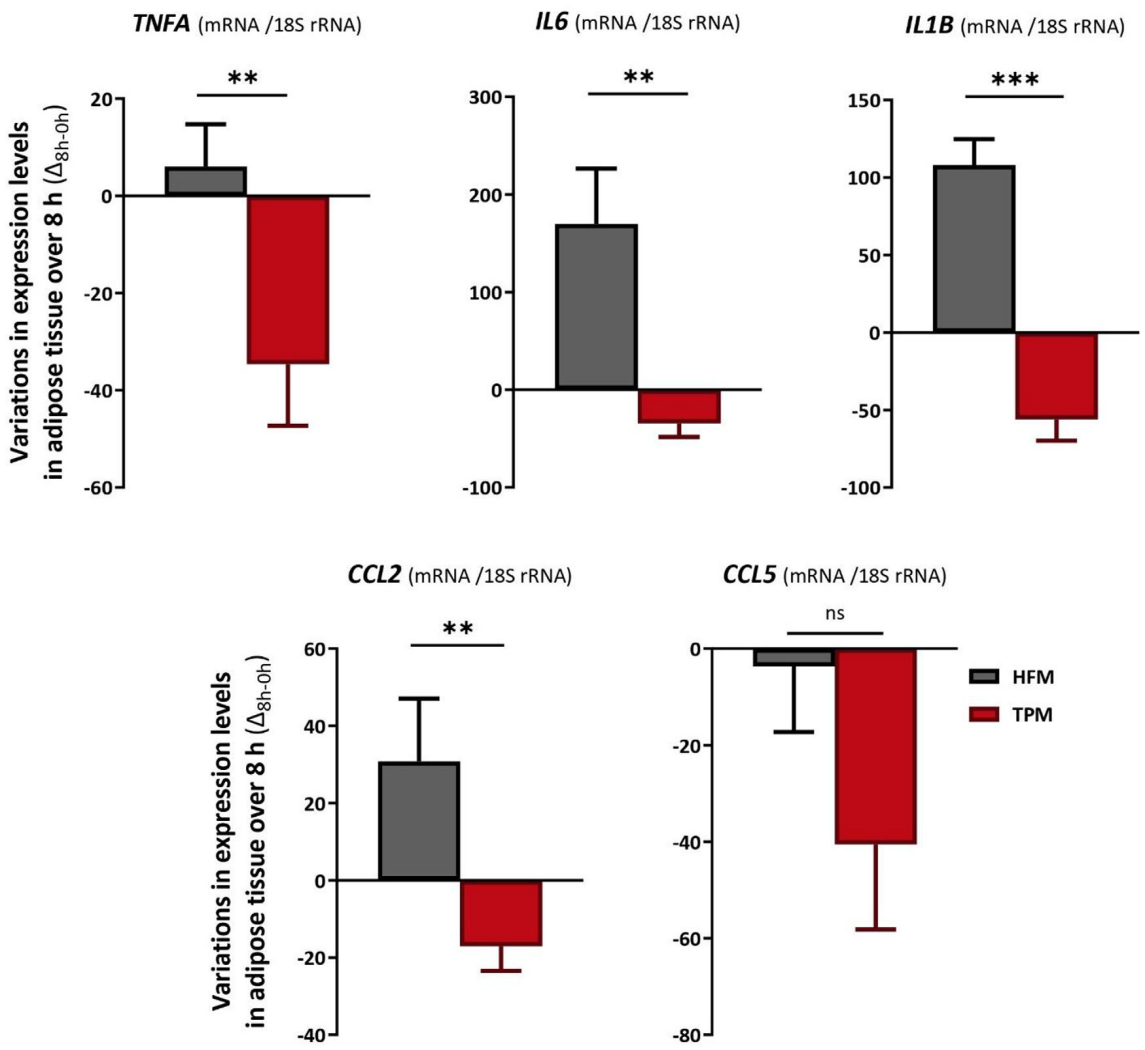
Inter‐group comparative analysis of postprandial variations in plasma concentrations of inflammatory markers (TPM vs. HFM). Mean ± SEM (biological replicates) of changes (Δ) in plasma concentrations between baseline (0 h) and postprandial (8 h). *p* values correspond to the ANOVA analysis of the GLM model that compared variations between TPM (red) (*n *= 39) and HFM (grey) (*n *= 39) groups; *p* < 0.05 indicates a significant difference, highlighted in bold. The difference between groups is indicated by *: *p* < 0.05; **: *p* < 0.01; ***: *p* < 0.001. The variation was calculated between each collection time (Δ_8h–0h_), according to the following calculation: (postprandial value—fasting value). *CCL2*, C‐C motif chemokine ligand 2; *CCL5*, C‐C motif chemokine ligand 5; HFM, high fat meal; *IL1B*, interleukin 1 beta; *IL6*, interleukin 6; *TNFA*, tumor necrosis factor alpha; TPM, tomato puree meal.

The GLM inter‐group comparison of the postprandial variation in adipose tissue inflammatory gene expression between the TPM and HFM is presented in Figure 4.

Firstly, variations in adipose tissue inflammatory gene expression between 0 and 8 h in the HFM were positive (right of axis 0) except for the inflammatory gene *CCL5*, while variations in adipose gene expression in the TPM were all negative (left of axis 0). Therefore, the expression of adipose tissue genes encoding inflammatory markers tended to increase postprandially for the HFM and decrease for the TPM. The expression of inflammatory gene encoding markers *TNFA*, *IL6*, *IL1B*, and *CCL2* in adipose tissue showed significantly reduced variations of 674.8% (−34.7 ± 12.7 vs. 6.0 ± 8.7, *p *= 0.0014), 120.4% (−34.6 ± 13.8 vs. 169.8 ± 56.8, *p* = 0.0018), 151.9% (−56.0 ± 13.8 vs. 108.0 ± 16.8, *p *= 4.978e‐07) and 155.5% (−17.1 ± 6.4 vs. 30.8 ± 16.3, *p *= 0.0061), between TPM and HFM, respectively. The expression of the inflammatory gene encoding *CCL5* in adipose tissue did not show a significant difference between variations in TPM and HFM (−40.6 ± 17.6 vs. −3.7 ± 13.6, *p *= 0.08764).

### 3.4 Correlation between Plasma and Adipose Tissue Inflammatory Markers

Analysis of the linear relationship of variations (Δ_8h–0h_) between plasma concentrations and adipose gene expression for each inflammatory marker and for each meal (TPM and HFM) revealed the existence of a positive and significant linear association between plasma concentrations and adipose gene expression of the inflammatory marker *IL1B* for the TPM (*β *= 2.0224, *R^2^ *= 0.1867, *p *= 0.0067; data not shown).

## 4 Discussion

### 4.1 Lycopene and Inflammation in Adipose Tissue

The present study assessed the benefit of tomato puree enrichment in an HFM on postprandial inflammation in healthy male adults. This study provides an advantage in investigating the acute effect of tomato puree enrichment on postprandial inflammation by evaluating both plasma inflammatory markers and gene expression in adipose tissue. So far, very limited clinical studies investigated the effect of lycopene or tomato product supplementation on postprandial inflammation [27, 34] and none have yet been published on gene expression in adipose tissue. However, it is known that lycopene, the main bioactive component of tomatoes, is mainly stored in adipose tissue, which represents about 60% of the total body reserves of lycopene [35, 36]. Therefore, lycopene accumulated at this level could exert a direct anti‐inflammatory action on cytokine signaling pathways [37, 38]. In line, the selection of pro‐inflammatory cytokines evaluated in this study was based on evidence that both are adipokines, that is, cytokines produced by adipose tissue. Moreover, these biomarkers are the most closely correlated with adipose tissue inflammation in the postprandial period [6]. The major finding was the observation that adding tomato puree to an HFM decreases both systemic inflammation and expression of inflammatory genes within adipose tissue. In the HFM group, inflammatory markers and gene expressions significantly increased after the meal. Conversely, TPM decreased both plasma inflammatory markers and gene expressions in adipose tissue. Furthermore, inter‐group analysis also showed that the inflammatory response was significantly lower in the TPM group compared to the HFM group. In line with our results, a clinical study conducted by Hurtado‐Barroso et al. reported a 20% decrease in plasma concentrations of TNF‐α and C‐reactive protein (CRP) markers by testing the effect of consuming a single dose of sofrito (240 g/70 kg) in 22 healthy male subjects for 3 days. No effect was found on the IL‐6 marker [39]. The findings of Watzl et al. evaluating the effect of tomato juice supplementation for 2 weeks on healthy subjects indicated an opposite effect, characterized by an increase in plasma concentrations of the marker TNF‐α [40]. Other clinical studies on tomato product supplementation have also shown decrease in plasma IL‐6 levels after ingestion [27, 28]. Decreases in plasma CCL‐2 concentrations were also observed in healthy individuals in other studies [25, 28, 41]. Additional research on subjects with metabolic syndrome or obesity showed a stronger effect of tomato product supplementation on inflammatory markers [42, 43, 44]. Tsitsimpikou et al. notably found a 10.2% decrease in the concentration of TNF‐α and IL‐6 [44]. The linear relationship between inflammatory markers at both plasma and adipose levels was tested using linear regression models. Through this approach, we identified a positive and significant linear relationship for the inflammatory marker IL‐1β. This result potentially suggests the specificity of the action of tomato puree enrichment on the inflammatory marker IL‐1β both at plasma and adipose tissue levels. This correlation could also involve a specificity in the mechanism of action of tomato metabolites on this biomarker.

### 4.2 Mechanism of Action of Lycopene and Synergistic Effect

From a mechanistic point of view, it is now well established that plasma lycopene concentration is a strong marker of tomato consumption and is associated with a significant reduction of low‐grade inflammation [15, 45, 46]. A recent review of the literature suggested that plasma lycopene depletion could be considered as one of the first indicators of low‐grade inflammation [41]. In agreement, research studies describing the in vitro mechanism of action of lycopene demonstrated that these effects might be due to its inhibitory action on the phosphorylation of p65 and IkappaB kinase (IκB), resulting in the deactivation of NF‐κB [47]. These findings were notably observed in adipocytes [18, 48, 49]. This was confirmed by a pre‐clinical study investigating the effect of a 12‐week supplementation with lycopene or tomato powder in addition to a high‐fat diet on obesity, inflammatory response, and associated metabolic disorders [17]. In this trial, the assessment of the inflammatory response was conducted by studying the expression of genes involved in inflammation as well as the circulating levels of associated cytokines. According to our study, the outcomes of this study highlight the beneficial effect of these supplementations on the inflammatory response by decreasing the expression of genes and circulating levels. These effects involve reduced phosphorylation of IκB and p65 proteins in adipose tissue, lowering inflammatory proteins and adiposity index [38, 50]. Other pre‐clinical studies have shown that lycopene or lycopene‐rich tomato supplements can reduce adipose tissue inflammation by inhibiting pro‐inflammatory gene expression or lowering plasma cytokine levels [16, 17, 50–55]. Besides lycopene, tomatoes are also a source of carotenoids (lutein, β‐carotene), vitamins C and E, and polyphenols, such as flavonoids and phenolic acids, known for their powerful anti‐inflammatory and antioxidant actions [19]. Although the beneficial effect of tomato product supplementation is usually attributed to lycopene, other tomato compounds also could play an essential synergetic role in anti‐inflammatory processes. Studies assessing the effect of lycopene isolated from its matrix offer the advantage of considering lycopene‐specific mechanisms of action and can be interesting in the context of lycopene supplementation. In promoting a varied and preventive diet, our study highlights the accessibility of a lycopene‐rich matrix containing other bioactive compounds, making it more widely available than supplements.

### 4.3 Limits and Perspectives

Several limitations should be noted. Although the meals were composed of the same quantities of ingredients, the addition of 100 g of tomato puree to the TPM increased energy, protein, carbohydrate, and fiber intake compared to the HFM. Even if this increase was modest, certain components of this diet are known to influence postprandial inflammation. For example, fiber is known to reduce inflammation, particularly postprandially [56], while carbohydrates have been studied for the opposite effect [1]. The design of this study did not adjust the macronutrient and micronutrient composition of the two meals voluntarily, to assess the impact of simply adding a portion of tomato puree to a HFM, taking into account the addition of other nutrients to the meal. Local anesthesia during adipose tissue biopsy may induce a local inflammatory response and potentially have an impact on gene expression data. However, this procedure was performed under the same experimental conditions for both types of meals, thus dismissing this potential effect. Additionally, this present study included 40 volunteers, exclusively male. While the unisex investigation helps to increase the power of the study and to limit the inter‐individual variability, it also represents a limitation of the study, as the findings cannot be generalized to women. Biological differences between men and women, such as hormonal variations, body composition, and metabolic rates, may lead to different responses to the intervention being studied. Therefore, the results may not fully capture the potential effects in a female population, limiting the broader applicability of the study's conclusions.

## 5 Conclusion

In conclusion, an enrichment of 100 g of tomato puree in a HFM reduced systemic postprandial inflammation in healthy subjects. This reduction was evident in both circulating cytokine levels and gene expressions in adipose tissue. This postprandial anti‐inflammatory effect would suggest a beneficial and preventive effect in the development of cardiometabolic diseases. Further long‐term studies are also needed in the context of a preventive diet, to establish a consensus on the anti‐inflammatory effect of tomato products.

## Author Contributions

The authors’ responsibilities were as follows—Jean‐François Landrier and Patrick Borel designed the research protocol; Djaffar Ould‐Ali and Julien Astier conducted research; Lea Sani analyzed data. Jean‐François Landrier had primary responsibility for the final content of the manuscript; Lea Sani and Jean‐François Landrier wrote the manuscript draft; and all authors read and approved the final manuscript.

## Conflicts of Interest

The authors declare no conflicts of interest.

## Data Availability

Data available on request from the authors.

## References

[1] E. C. E. Meessen , M. V. Warmbrunn , M. Nieuwdorp , and M. R. Soeters , “Human Postprandial Nutrient Metabolism and Low‐Grade Inflammation: A Narrative Review,” Nutrients 11, no. 12 (2019): 3000, 10.3390/nu11123000. 31817857 PMC6950246

[2] B. Burton‐Freeman , “Postprandial Metabolic Events and Fruit‐Derived Phenolics: A Review of the Science,” British Journal of Nutrition 104, no. S3 (2010): S1–S14, 10.1017/S0007114510003909. 20955646

[3] B. K. S. Silveira , T. M. S. Oliveira , P. A. Andrade , H. H. M. Hermsdorff , C. d. O. B. Rosa , and S. d. C. C. Franceschini , “Dietary Pattern and Macronutrients Profile on the Variation of Inflammatory Biomarkers: Scientific Update,” Cardiology Research and Practice 2018 (2018): 4762575, 10.1155/2018/4762575. 29725543 PMC5872610

[4] G. Pang , J. Xie , Q. Chen , and Z. Hu , “Energy Intake, Metabolic Homeostasis, and Human Health,” Food Science and Human Wellness 3, no. 3 (2014): 89–103, 10.1016/j.fshw.2015.01.001.

[5] E. Gouranton , C. Thabuis , C. Riollet , et al., “Lycopene Inhibits Proinflammatory Cytokine and Chemokine Expression in Adipose Tissue,” Journal of Nutritional Biochemistry 22, no. 7 (2011): 642–648, 10.1016/j.jnutbio.2010.04.016. 20952175

[6] M. Herieka and C. Erridge , “High‐Fat Meal Induced Postprandial Inflammation,” Molecular Nutrition & Food Research 58, no. 1 (2014): 136–145, 10.1002/mnfr.201300104. 23847095

[7] C. Tsigalou , T. Konstantinidis , A. Paraschaki , E. Stavropoulou , C. Voidarou , and E. Bezirtzoglou , “Mediterranean Diet as a Tool to Combat Inflammation and Chronic Diseases. An Overview,” Biomedicines 8, no. 7 (2020): 201, 10.3390/biomedicines8070201. 32650619 PMC7400632

[8] R. J. Widmer , A. J. Flammer , L. O. Lerman , and A. Lerman , “The Mediterranean Diet, Its Components, and Cardiovascular Disease,” American Journal of Medicine 128, no. 3 (2015): 229–238, 10.1016/j.amjmed.2014.10.014. 25447615 PMC4339461

[9] M. A. Martínez‐González , A. Gea , and M. Ruiz‐Canela , “The Mediterranean Diet and Cardiovascular Health,” Circulation Research 124, no. 5 (2019): 779–798, 10.1161/CIRCRESAHA.118.313348. 30817261

[10] V. Rosato , N. J. Temple , C. La Vecchia , G. Castellan , A. Tavani , and V. Guercio , “Mediterranean Diet and Cardiovascular Disease: A Systematic Review and Meta‐Analysis of Observational Studies,” European Journal of Nutrition 58, no. 1 (2019): 173–191, 10.1007/s00394-017-1582-0. 29177567

[11] D. Sleiman , M. R. Al‐Badri , and S. T. Azar , “Effect of Mediterranean Diet in Diabetes Control and Cardiovascular Risk Modification: A Systematic Review,” Front Public Health 3 (2015): 69. 25973415 10.3389/fpubh.2015.00069 PMC4411995

[12] R. Mirondo and S. Barringer , “Improvement of Flavor and Viscosity in Hot and Cold Break Tomato Juice and Sauce by Peel Removal,” Journal of Food Science 80, no. 1 (2015): S171–S179, 10.1111/1750-3841.12725. 25603846

[13] K. Canene‐Adams , J. K. Campbell , S. Zaripheh , and E. H. Jeffery , “The Tomato as a Functional Food,” Journal of Nutrition 135, no. 5 (2005): 1226–1230, 10.1093/jn/135.5.1226. 15867308

[14] R. Romano , L. De Luca , N. Manzo , F. Pizzolongo , and A. Aiello , “A New Type of Tomato Puree With High Content of Bioactive Compounds From 100% Whole Fruit,” Journal of Food Science 85, no. 10 (2020): 3264–3272, 10.1111/1750-3841.15423. 32885436

[15] H. M. Cheng , G. Koutsidis , J. K. Lodge , A. Ashor , M. Siervo , and J. Lara , “Tomato and Lycopene Supplementation and Cardiovascular Risk Factors: A Systematic Review and Meta‐Analysis,” Atherosclerosis 257 (2017): 100–108, 10.1016/j.atherosclerosis.2017.01.009. 28129549

[16] R. de A. M. Luvizotto , A. F. Nascimento , E. Imaizumi , et al., “Lycopene Supplementation Modulates Plasma Concentrations and Epididymal Adipose Tissue mRNA of Leptin, Resistin and IL‐6 in Diet‐Induced Obese Rats,” British Journal of Nutrition 110, no. 10 (2013): 1803–1809, 10.1017/S0007114513001256. 23632237

[17] S. Fenni , H. Hammou , J. Astier , et al., “Lycopene and Tomato Powder Supplementation Similarly Inhibit High‐Fat Diet Induced Obesity, Inflammatory Response, and Associated Metabolic Disorders,” Molecular Nutrition & Food Research 61, no. 9 (2017): 1601083, 10.1002/mnfr.201601083. 28267248

[18] S. Fenni , J. Astier , L. Bonnet , et al., “(all‐E)‐ and (5Z)‐(all‐E)‐ and (5Z)‐Lycopene Display Similar Biological Effects on Adipocytes,” Molecular Nutrition & Food Research 63, no. 5 (2019): 1800788, 10.1002/mnfr.201800788. 30512227

[19] A. Raiola , M. M. Rigano , R. Calafiore , L. Frusciante , and A. Barone , “Enhancing the Health‐Promoting Effects of Tomato Fruit for Biofortified Food,” Mediators of Inflammation 2014 (2014): 139873, 10.1155/2014/139873. 24744504 PMC3972926

[20] K. Jacob , M. J. Periago , V. Böhm , and G. R. Berruezo , “Influence of Lycopene and Vitamin C From Tomato Juice on Biomarkers of Oxidative Stress and Inflammation,” British Journal of Nutrition 99, no. 1 (2008): 137–146, 10.1017/S0007114507791894. 17640421

[21] S. Agarwal and A. V. Rao , “Tomato Lycopene and Low Density Lipoprotein Oxidation: A Human Dietary Intervention Study,” Lipids 33, no. 10 (1998): 981–984, 10.1007/s11745-998-0295-6. 9832077

[22] M.‐L. Silaste , G. Alfthan , A. Aro , Y. Antero Kesäniemi , and S. Hörkkö , “Tomato Juice Decreases LDL Cholesterol Levels and Increases LDL Resistance to Oxidation,” British Journal of Nutrition 98, no. 6 (2007): 1251–1258, 10.1017/S0007114507787445. 17617941

[23] S. Devaraj , S. Mathur , A. Basu , et al., “A Dose‐Response Study on the Effects of Purified Lycopene Supplementation on Biomarkers of Oxidative Stress,” Journal of the American College of Nutrition 27, no. 2 (2008): 267–273. 18689558 10.1080/07315724.2008.10719699 PMC2677959

[24] C. W. Hadley , S. K. Clinton , and S. J. Schwartz , “The Consumption of Processed Tomato Products Enhances Plasma Lycopene Concentrations in Association With a Reduced Lipoprotein Sensitivity to Oxidative Damage,” Journal of Nutrition 133, no. 3 (2003): 727–732, 10.1093/jn/133.3.727. 12612144

[25] Y.‐F. Li , Y.‐Y. Chang , H.‐C. Huang , Y.‐C. Wu , M.‐D. Yang , and P.‐M. Chao , “Tomato Juice Supplementation in Young Women Reduces Inflammatory Adipokine Levels Independently of Body Fat Reduction,” Nutrition 31, no. 5 (2015): 691–696, 10.1016/j.nut.2014.11.008. 25837214

[26] B. M. Burton‐Freeman and H. D. Sesso , “Whole Food Versus Supplement: Comparing the Clinical Evidence of Tomato Intake and Lycopene Supplementation on Cardiovascular Risk Factors,” Advances in Nutrition 5, no. 5 (2014): 457–485, 10.3945/an.114.005231. 25469376 PMC4188219

[27] B. Burton‐Freeman , J. Talbot , E. Park , S. Krishnankutty , and I. Edirisinghe , “Protective Activity of Processed Tomato Products on Postprandial Oxidation and Inflammation: A Clinical Trial in Healthy Weight Men and Women,” Molecular Nutrition & Food Research 56, no. 4 (2012): 622–631, 10.1002/mnfr.201100649. 22331646

[28] P. Valderas‐Martinez , G. Chiva‐Blanch , R. Casas , et al., “Tomato Sauce Enriched With Olive Oil Exerts Greater Effects on Cardiovascular Disease Risk Factors than Raw Tomato and Tomato Sauce: A Randomized Trial,” Nutrients 8, no. 3 (2016): 170, 10.3390/nu8030170. 26999197 PMC4808898

[29] https://ciqual.anses.fr.

[30] P. Borel , C. Desmarchelier , M. Nowicki , and R. Bott , “Lycopene Bioavailability Is Associated With a Combination of Genetic Variants,” Free Radical Biology and Medicine 83 (2015): 238–244, 10.1016/j.freeradbiomed.2015.02.033. 25772008

[31] J.‐F. Landrier , C. Malezet‐Desmoulins , E. Reboul , A. Marie Lorec , M. Josèphe Amiot , and P. Borel , “Comparison of Different Vehicles to Study the Effect of Tocopherols on Gene Expression in Intestinal Cells,” Free Radical Research 42, no. 5 (2008): 523–530, 10.1080/10715760802098859. 18484416

[32] K. J. Livak and T. D. Schmittgen , “Analysis of Relative Gene Expression Data Using Real‐Time Quantitative PCR and the 2−ΔΔCT Method,” Methods 25, no. 4 (2001): 402–408. 11846609 10.1006/meth.2001.1262

[33] Y. Sisman , “Outlier Measurement Analysis With the Robust Estimation,” Scientific Research and Essays 5, no. 7 (2010): 668–678.

[34] S. G. Denniss , T. D. Haffner , J. T. Kroetsch , S. R. Davidson , J. W. Rush , and R. L. Hughson , “Effect of Short‐Term Lycopene Supplementation and Postprandial Dyslipidemia on Plasma Antioxidants and Biomarkers of Endothelial Health in Young, Healthy Individuals,” Vascular Health and Risk Management 4, no. 1 (2008): 213–222. 18629373 10.2147/vhrm.2008.04.01.213 PMC2464768

[35] V. Böhm , G. Lietz , B. Olmedilla‐Alonso , et al., “From Carotenoid Intake to Carotenoid Blood and Tissue Concentrations—Implications for Dietary Intake Recommendations,” Nutrition Reviews 79, no. 5 (2021): 544–573, 10.1093/nutrit/nuaa008. 32766681 PMC8025354

[36] N. E. Moran , J. W. Erdman , and S. K. Clinton , “Complex Interactions Between Dietary and Genetic Factors Impact Lycopene Metabolism and Distribution,” Archives of Biochemistry and Biophysics 539, no. 2 (2013): 171–180, 10.1016/j.abb.2013.06.017. 23845854 PMC3818361

[37] N. Markovits , A. B. Amotz , and Y. Levy , “The Effect of Tomato‐Derived Lycopene on Low Carotenoids and Enhanced Systemic Inflammation and Oxidation in Severe Obesity,” The Israel Medical Association Journal 11, no. 10 (2009): 598–601. 20077945

[38] J.‐F. Landrier , T. Breniere , L. Sani , C. Desmarchelier , L. Mounien , and P. Borel , “Effect of Tomato, Tomato‐Derived Products and Lycopene on Metabolic Inflammation: From Epidemiological Data to Molecular Mechanisms,” Nutrition Research Reviews 38, no. 1 (2023): 95–111, 10.1017/S095442242300029X. 38105560

[39] S. Hurtado‐Barroso , M. Martínez‐Huélamo , J. F. Rinaldi de Alvarenga , et al., “Acute Effect of a Single Dose of Tomato Sofrito on Plasmatic Inflammatory Biomarkers in Healthy Men,” Nutrients 11, no. 4 (2019): 851, 10.3390/nu11040851. 30991720 PMC6520770

[40] B. Watzl , A. Bub , K. Briviba , and G. Rechkemmer , “Supplementation of a Low‐Carotenoid Diet With Tomato or Carrot Juice Modulates Immune Functions in Healthy Men,” Annals of Nutrition & Metabolism 47, no. 6 (2003): 255–261, 10.1159/000072397. 14520020

[41] C. Sánchez‐Moreno , M. P. Cano , B. de Ancos , et al., “Mediterranean Vegetable Soup Consumption Increases Plasma Vitamin C and Decreases F2‐Isoprostanes, Prostaglandin E2 and Monocyte Chemotactic Protein‐1 in Healthy Humans,” Journal of Nutritional Biochemistry 17, no. 3 (2006): 183–189, 10.1016/j.jnutbio.2005.07.001. 16169205

[42] M. Ghavipour , A. Saedisomeolia , M. Djalali , et al., “Tomato Juice Consumption Reduces Systemic Inflammation in Overweight and Obese Females,” British Journal of Nutrition 109, no. 11 (2013): 2031–2035, 10.1017/S0007114512004278. 23069270

[43] M. Colmán‐Martínez , M. Martínez‐Huélamo , P. Valderas‐Martínez , et al., “trans‐Lycopene From Tomato Juice Attenuates Inflammatory Biomarkers in Human Plasma Samples: An Intervention Trial,” Molecular Nutrition & Food Research 61, no. 11 (2017): 1600993, 10.1002/mnfr.201600993. 28688174

[44] C. Tsitsimpikou , K. Tsarouhas , N. Kioukia‐Fougia , et al., “Dietary Supplementation With Tomato‐Juice in Patients With Metabolic Syndrome: A Suggestion to Alleviate Detrimental Clinical Factors,” Food and Chemical Toxicology 74 (2014): 9–13, 10.1016/j.fct.2014.08.014. 25194627

[45] P. C. Calder , N. Ahluwalia , F. Brouns , et al., “Dietary Factors and Low‐Grade Inflammation in Relation to Overweight and Obesity,” British Journal of Nutrition 106, no. S3 (2011): S5–S78, 10.1017/S0007114511005460. 22133051

[46] A. V. Rao , G. L. Young , and L. G. Rao , Lycopene and Tomatoes in Human Nutrition and Health (CRC Press, 2018).

[47] H. P. van Steenwijk , A. Bast , and A. de Boer , “The Role of Circulating Lycopene in Low‐Grade Chronic Inflammation: A Systematic Review of the Literature,” Molecules 25, no. 19 (2020): 4378, 10.3390/molecules25194378. 32977711 PMC7582666

[48] F. Tourniaire , B. Romier‐Crouzet , J. H. Lee , et al., “Chemokine Expression in Inflamed Adipose Tissue Is Mainly Mediated by NF‐κB,” PLoS ONE 8, no. 6 (2013): 66515, 10.1371/journal.pone.0066515. PMC3688928 23824685

[49] J. Marcotorchino , B. Romier , E. Gouranton , et al., “Lycopene Attenuates LPS‐Induced TNF‐α Secretion in Macrophages and Inflammatory Markers in Adipocytes Exposed to Macrophage‐Conditioned Media,” Molecular Nutrition & Food Research 56, no. 5 (2012): 725–732, 10.1002/mnfr.201100623. 22648619

[50] J. Wang , Y. Suo , J. Zhang , et al., “Lycopene Supplementation Attenuates Western Diet‐Induced Body Weight Gain through Increasing the Expressions of Thermogenic/Mitochondrial Functional Genes and Improving Insulin Resistance in the Adipose Tissue of Obese Mice,” Journal of Nutritional Biochemistry 69 (2019): 63–72, 10.1016/j.jnutbio.2019.03.008. 31060024

[51] I. H. Bahcecioglu , N. Kuzu , K. Metin , et al., “Lycopene Prevents Development of Steatohepatitis in Experimental Nonalcoholic Steatohepatitis Model Induced by High‐Fat Diet,” Veterinary Medicine International 2010 (2010): 262179, 10.4061/2010/262179. 20953409 PMC2952801

[52] J. Wang , Q. Zou , Y. Suo , et al., “Lycopene Ameliorates Systemic Inflammation‐Induced Synaptic Dysfunction via Improving Insulin Resistance and Mitochondrial Dysfunction in the Liver–Brain Axis,” Food & Function 10, no. 4 (2019): 2125–2137, 10.1039/C8FO02460J. 30924473

[53] C.‐C. Li , C. Liu , M. Fu , et al., “Tomato Powder Inhibits Hepatic Steatosis and Inflammation Potentially through Restoring SIRT1 Activity and Adiponectin Function Independent of Carotenoid Cleavage Enzymes in Mice,” Molecular Nutrition & Food Research 62, no. 8 (2018): 1700738, 10.1002/mnfr.201700738. 29266812

[54] G. Chen , Y. Ni , N. Nagata , et al., “Lycopene Alleviates Obesity‐Induced Inflammation and Insulin Resistance by Regulating M1/M2 Status of Macrophages,” Molecular Nutrition & Food Research 63, no. 21 (2019): 1900602, 10.1002/mnfr.201900602. 31408586

[55] T. Breniere , L. Bournot , F. Sicard , et al., “Tomato Genotype but Not Crop Water Deficit Matters for Tomato Health Benefits in Diet‐Induced Obesity of C57BL/6JRj Male Mice,” Food Research International 188 (2024): 114512, 10.1016/j.foodres.2024.114512. 38823883

[56] E. J. Reverri , J. M. Randolph , F. M. Steinberg , C. T. Kappagoda , I. Edirisinghe , and B. M. Burton‐Freeman , “Black Beans, Fiber, and Antioxidant Capacity Pilot Study: Examination of Whole Foods vs. Functional Components on Postprandial Metabolic, Oxidative Stress, and Inflammation in Adults With Metabolic Syndrome,” Nutrients 7, no. 8 (2015): 6139–6154, 10.3390/nu7085273. 26225995 PMC4555112

